# Cerebrospinal fluid procalcitonin and neutrophil percentage: a combined biomarker for differentiating bacterial from tuberculous meningitis in antibiotic-pretreated patients

**DOI:** 10.3389/fcimb.2026.1825236

**Published:** 2026-06-23

**Authors:** Yaoyao Zhang, Anxin Liang, Xiaona Li, Xin Zhang, Xin Guo, Zhongqing Sun, Kejian Wu, Wen Li

**Affiliations:** 1 Department of Neurology, Xijing Hospital, Fourth Military Medical University, Xi’an, China; 2 Department of Mathematics and Physics, School of Basic Medicine, Fourth Military Medical University, Xi’an, China

**Keywords:** bacterial meningitis, cerebrospinal fluid, neutrophil percentage, procalcitonin, prognosis, tuberculous meningitis

## Abstract

**Background:**

Distinguishing between bacterial meningitis (BM) and tuberculous meningitis (TBM) is a persistent clinical dilemma, further complicated by widespread empirical antibiotic use. This study evaluated the diagnostic and prognostic utility of cerebrospinal fluid procalcitonin (CSF-PCT) and other laboratory markers, analyzing all enrolled patients irrespective of prior antibiotic exposure.

**Methods:**

A prospective cohort analysis was conducted from September 2014 to September 2025, consecutively enrolling 125 BM patients and 56 TBM patients. Patients were included regardless of their pre - admission antibiotic treatment history. Demographic and laboratory parameters upon admission were analyzed. Receiver operating characteristic and multivariate logistic regression analyses were employed to identify biomarkers for differentiating BM from TBM and predictors of unfavorable outcomes (Glasgow Coma Scale score ≤8 at discharge) in BM.

**Results:**

The majority (89.5%) of patients had prior antibiotics. CSF-PCT and CSF neutrophil percentage demonstrated comparable efficacy in distinguishing BM from TBM, with area under the curve (AUC) values of 0.772 and 0.786, respectively. A combined model integrating both biomarkers achieved superior diagnostic performance (AUC = 0.849), significantly outperforming either marker alone (both *P* < 0.01). Multiple logistic regression analysis identified CSF-PCT >0.08 ng/mL (OR = 3.816), CSF neutrophil percentage >39% (OR = 20.253), and serum interleukin-6 (IL-6) >17.65 pg/mL (OR = 6.507) as independent discriminators of BM from TBM. Additionally, elevated CSF-PCT (>0.271 ng/mL) was the sole independent predictor for short-term adverse outcomes in BM (adjusted OR = 7.111, AUC = 0.727).

**Conclusion:**

The combination of CSF-PCT and CSF neutrophil percentage serves as a reliable biomarker for differentiating BM from TBM. CSF-PCT stands as an independent risk factor for unfavorable short-term outcomes in BM. These findings confirm the clinical utility of these biomarkers in early diagnosis and risk stratification, which remains effective in the routine clinical setting where patients commonly present with a history of antibiotic use.

## Introduction

Bacterial meningitis (BM) constitutes a significant global health burden, despite the increased vaccination coverage against common meningeal pathogens (van de Beek et al., 2021; van Ettekoven et al., 2024). The incidence rates range from approximately 0.9 cases per 100,000 individuals annually in high-income countries to 80 cases per 100,000 in low- and middle-income countries (Hasbun, 2022). The mortality rates vary among different populations and countries, spanning from 6% to 58% (Collaborators G. B. D. C. O. D., 2017; Collaborators G. B. D. M. A. R., 2023). Up to 25% of survivors develop chronic neurological sequelae (Lucas et al., 2016). Timely identification of BM remains crucial yet challenging due to the non-specific clinical manifestations, especially when differentiating it from tuberculous meningitis (TBM) (Manyelo et al., 2021; Paing et al., 2024). To address this challenge, there is a pressing need for reliable biomarkers and advanced detection technologies.

Although cerebrospinal fluid (CSF) culture remains the microbiological gold standard, its sensitivity drops below 50% after antibiotic administration (van de Beek et al., 2016). Serum procalcitonin (PCT), a peptide precursor of the hormone calcitonin, has been shown to be a diagnostic biomarker for BM in many studies (Dubos et al., 2008; Shen et al., 2015; Li et al., 2017; Sager et al., 2017; Li et al., 2022). However, these studies are limited to serum analysis or primarily focus on diagnostic accuracy rather than prognostication. Additionally, most studies excluded patients who had received antibiotic therapy before serum PCT testing. Building on this, our prior research demonstrated that CSF-PCT is a more effective diagnostic biomarker for BM, even after empirical antibiotic treatment, particularly in Gram-negative BM (Li et al., 2017; Li et al., 2019; Li et al., 2025). Nevertheless, its utility in distinguishing BM from TBM, as well as its correlation with discharge outcomes, still requires clarification.

To address this, we conducted a prospectively designed study to evaluate the diagnostic efficacy of CSF-PCT in distinguishing BM from TBM in an enlarged sample cohort and to assess its prognostic significance, with particular attention paid to its performance in patients with previous empirical antibiotic therapy.

## Methods

### Study design and setting

This prospective observational cohort study was conducted in the Neurological Intensive Care Unit (NICU) of Xijing Hospital, a tertiary academic medical institution in Shaanxi Province, China. The study consecutively enrolled patients diagnosed with acute bacterial or tuberculous meningitis. Written informed consent was obtained from each participant or their legal guardian. The study protocol was approved by the Ethics Committee of Xijing Hospital (KY20140916-3) and was carried out in accordance with Chinese law and the most recent version of the Declaration of Helsinki.

### Patients

From September 2014 to September 2025, consecutive patients aged ≥13 years with acute bacterial or tuberculous (definite or probable) meningitis were included. Most patients were transferred from primary care centers or the emergency department of our hospital, where they had received preliminary treatment, such as empirical antibiotic therapy (third-generation cephalosporins ± vancomycin). BM was diagnosed based on typical clinical features and at least one of the following: 1) a positive CSF culture; 2) bacteria identified through CSF Gram staining, smear, polymerase chain reaction (PCR), or next-generation sequencing (NGS); or 3) a CSF leukocyte count of ≥500/mm^3^ with rapid clinical improvement following the initiation of antibiotic therapy (Klein, 2014). Definite TBM was established when patients exhibited one of the following results: 1) a positive CSF smear microscopy for acid-fast bacilli; 2) a positive CSF culture, or PCR for *Mycobacterium tuberculosis*; or 3) a positive Xpert MTB/RIF assay of CSF. Patients were diagnosed with probable TBM if they fulfilled the following criteria: 1) scored ≥10 points on the diagnostic scoring system in the absence of cerebral imaging, or ≥12 points with cerebral imaging (with at least 2 points originating from imaging or CSF studies); and 2) the condition improved significantly after antituberculosis therapy (Marais et al., 2010). The final diagnosis for each patient was determined by two experienced neurologists, who reached a consensus based on the aforementioned criteria. Notably, the diagnoses were established by neurologists based on standard clinical and microbiological criteria, and that CSF PCT was analyzed as a research biomarker without being incorporated into the diagnostic algorithm. Patients without CSF-PCT measurement and those with concurrent viral, fungal, or tuberculous infections were excluded. Patients with previous empirical antibiotic treatment were not excluded regardless of the type, dosage, or administration route.

### Data measurements and collection

All eligible patients underwent lumbar puncture upon admission if they had no contraindications to the procedure. The levels of CSF and serum PCT, interleukin-6 (IL-6), leukocyte counts, protein, neutrophil percentage, and glucose were measured. The concentrations of PCT were measured using an electrochemiluminescence immunoassay analyzer (Roche, Basel).

Demographic data, clinical features upon admission, treatment details, comorbidities, complications, and the length of NICU stay were collected. Key time intervals were also recorded, including the period from the onset of prodromal symptoms to lumbar puncture and to the initial CSF-PCT measurement. Specifically, we defined the time to antibiotic initiation as the time from the onset of symptoms to the initial administration of antibiotics; the duration of antibiotic treatment as the time from the first dose to CSF-PCT detection; and the time to PCT measurement (abbreviated as PCT time) as the time from the onset of prodromal symptoms to the initial CSF - PCT measurement.

### Definition of outcomes

Clinical outcomes were independently assessed at discharge by a neurologist blinded to all clinical data. The Glasgow Coma Scale (GCS) score was used to classify outcomes: unfavorable (GCS ≤ 8) and favorable (GCS > 8).

### Statistical analysis

Continuous variables were presented as mean ± standard deviation (for normal distribution) or median with interquartile range (IQR) (for non-normal distribution). Categorical variables were summarized as counts and percentages. Group comparisons for categorical variables were performed using the χ² test or Fisher’s exact test, as appropriate. Continuous variables between groups were compared using the Student’s *t-*test (for normal distribution) or Mann–Whitney *U* test (for non-normal distribution).

Univariate analyses were initially conducted to identify factors that distinguish BM from TBM and to determine risk predictors of unfavorable outcomes among BM cases. Variables with p < 0.10 were then included in a backward stepwise multivariate logistic regression model to further establish independent diagnostic biomarkers or prognostic predictors.

Optimal cutoff values for continuous biomarkers were determined using receiver operating characteristic (ROC) curve analysis. The results were reported as the area under the curve (AUC), 95% confidence interval (CI), and standard error (SE). For multivariate modeling, continuous variables were dichotomized based on these cutoffs. A two-tailed *P* < 0.05 was considered statistically significant. All statistical analyses were performed using SPSS version 24.0 (IBM Corp., Armonk) and MedCalc version 22.7 (MedCalc, Ostend).

## Results

**Figure 1:**
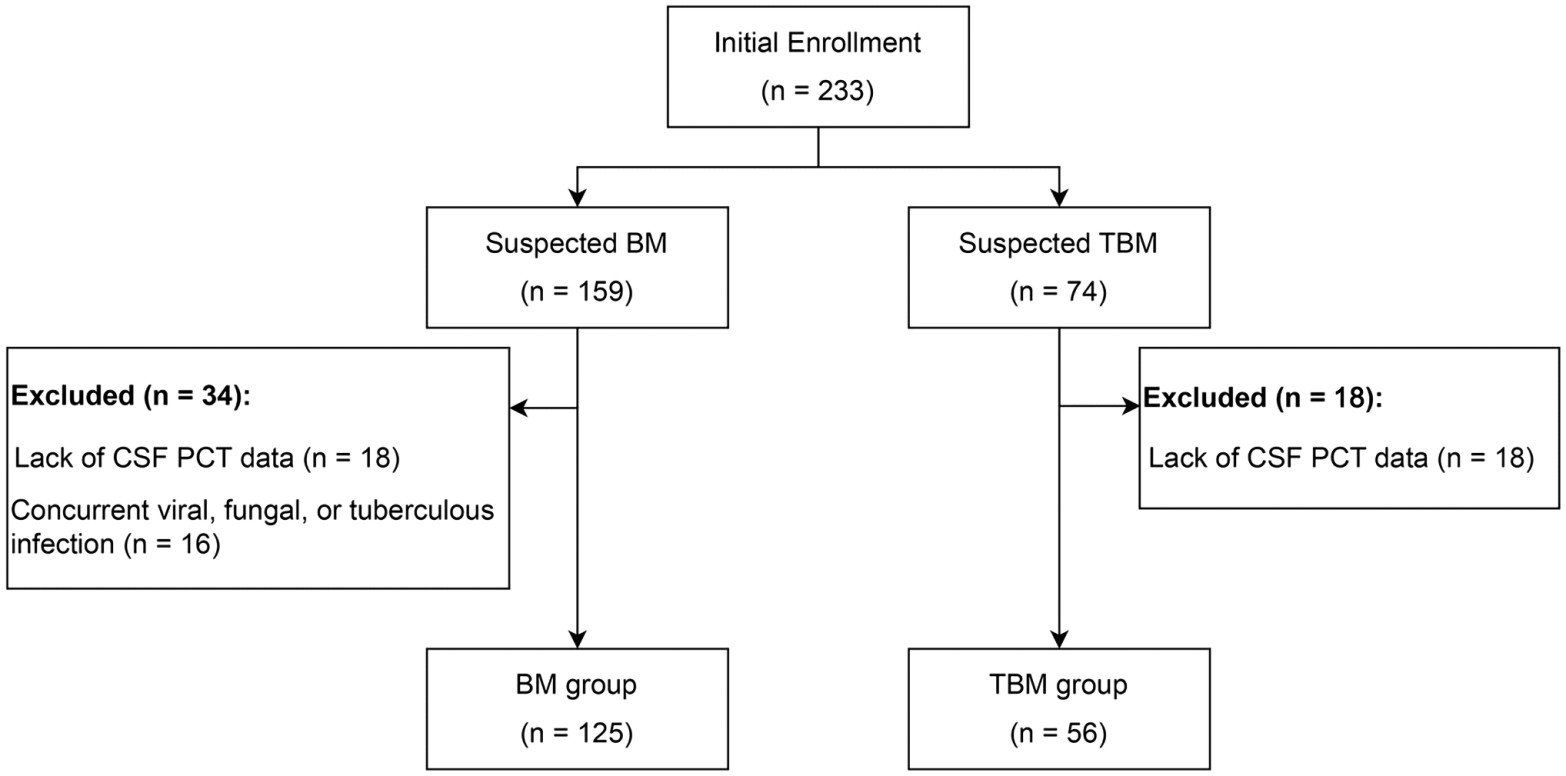
Flow chart of the study.

A total of 125 patients diagnosed with BM and 56 diagnosed with TBM were finally enrolled in this study (**Figure 1**). Among the 125 BM patients, 75 exhibited confirmed bacterial etiology [positive CSF culture, smear, PCR, or NGS results]. The identified pathogens included *Streptococcus* (n = 19), *Staphylococcus* (n = 14), *Klebsiella* (n = 11), *Listeria* (n = 3), *Enterococcus* (n = 3), and other bacterial species (n = 25) (**Supplementary Table 1**). Among the 56 TBM patients, 29 were microbiologically confirmed (definite TBM) via positive CSF culture, smear, or PCR for *M. tuberculosis*, and the remaining 27 (48.2%) were categorized as probable TBM. The majority (89.5%) of enrolled patients had prior antibiotics.

### Baseline characteristics and laboratory findings

The baseline characteristics and laboratory findings of patients with BM and TBM were summarized and compared (**Table 1**). No significant differences were observed between the two groups in terms of demographic profile (all *P* > 0.05). On admission, BM patients presented with lower GCS scores (*P* = 0.022) and higher Acute Physiology and Chronic Health Evaluation II (APACHE II) scores (*P* = 0.048) than the TBM cases. However, GCS scores at discharge showed no significant difference between the groups (*P* = 0.285). Notably, in-hospital mortality was significantly higher in the BM group (9.6% vs 0%, *P* = 0.019). Detail baseline clinical characteristics between the two groups are presented in **Supplementary Table 2**.

**Table 1:** Comparison of demographic and laboratory findings between patients with bacterial meningitis (BM) and tuberculous meningitis (TBM).

Parameters	BM (n = 125)	TBM (n = 56)	*P* value
Demographic profile			
Age, median (IQR), y^a^	46.0 (32.5, 55.0)	38.5 (25.0, 51.8)	0.055
Gender, male, n (%)^b^	94 (75.2)	35 (62.5)	0.081
Diabetes mellitus, n (%)^b^	15 (12.0)	2 (3.6)	0.072
CSF features (IQR)^a^			
PCT, median, ng/mL	0.15 (0.09, 0.46)	0.08 (0.05, 0.12)	**< 0.001**
IL-6, median, pg/ mL	830.90 (83.82, 5000.00)	195.80 (26.25, 1383.25)	**0.012**
Leukocyte, cells/mm^3^	259.0 (97.5, 1533.0)	111 (57, 254)	**< 0.001**
Neutrophil percentage, %	70 (20, 87)	17.5 (1.0, 28.0)	**< 0.001**
Protein, g/L	1.40 (0.73, 2.89)	1.14 (0.61, 2.08)	0.124
Glucose, mg/dL	46.62 (26.74, 60.84)	39.99 (26.33, 57.65)	0.307
Opening pressure, mmH_2_O	185 (145, 260)	248 (160, 330)	**0.007**
Closing pressure, mmH_2_O	100 (70, 150)	110 (80, 180)	0.144
Serum features			
PCT, ng/mL, (IQR)^a^	0.34 (0.09, 2.13)	0.10 (0.04, 0.25)	**< 0.001**
IL-6, median, pg/mL, (IQR)^a^	19.08 (8.11, 55.16)	10.24 (5.49, 17.09)	**< 0.001**
Leukocyte, ×10^6^ /L, (IQR)^a^	11600 (7945, 15010)	8905 (6305, 11275)	**< 0.001**
Albumin, g/L, (mean ± standard deviation (mean ± SD): $\left(\bar{X}\pm s\right)$)^c^	34.42±5.42	37.33±5.15	**< 0.001**
Glucose, mg/dL, (IQR)^a^	126.00 (98.10, 148.50)	104.40 (80.34, 130.62)	**0.003**
CSF to serum ratio (IQR)			
CSF/serum PCT ratio	0.53 (0.14, 1.49)	0.72 (0.41, 1.32)	0.129
CSF/serum IL-6 ratio	23.82 (2.11, 107.80)	24.98 (2.22, 122.86)	0.974
CSF/serum glucose ratio	0.37 (0.21, 0.52)	0.36 (0.22, 0.56)	0.563
Clinical profile			
GCS score on admission, median (IQR)^a^	10 (6, 15)	14 (8, 15)	**0.022**
APACHE II score on admission, median (IQR)^a^	9 (5, 16)	7 (3, 12)	**0.048**
GCS score at discharge, median (IQR)^a^	15 (10, 15)	15 (13, 15)	0.285
Death, n (%)^b^	12 (9.6)	0	**0.019**

IQR, interquartile range; CSF, cerebrospinal fluid; PCT, procalcitonin; IL-6, interleukin-6; GCS, Glasgow Coma Scale; APACHE II, Acute Physiology and Chronic Health Evaluation II; .

^a^ Mann-Whitney *U* test. ^b^*χ^2^* or Fisher exact test. ^c^Student’s *t* test.Bold values indicated statistically significant differences.

CSF analyses revealed that the BM group exhibited significantly higher levels of PCT (*P* < 0.001), IL-6 concentrations (*P* = 0.012), total leukocyte counts (*P* < 0.001), and neutrophil percentage (*P* < 0.001). In contrast, the opening pressure was significantly higher in the TBM group (*P* = 0.007). There were no significant differences in CSF protein (*P* = 0.124) and glucose (*P* = 0.307) levels between the two groups. Serum biomarkers similarly demonstrated elevated levels of PCT, IL-6, total leukocyte counts, and blood glucose in BM patients, whereas TBM patients had significantly higher serum albumin levels (all *P* < 0.05). Analysis of the CSF-to-serum ratios for PCT (*P* = 0.129), IL-6 (*P* = 0.974), and glucose (*P* = 0.563) levels revealed no statistically significant differences.

### Diagnostic performance of biomarkers for differentiating BM from TBM

**Figure 2:**
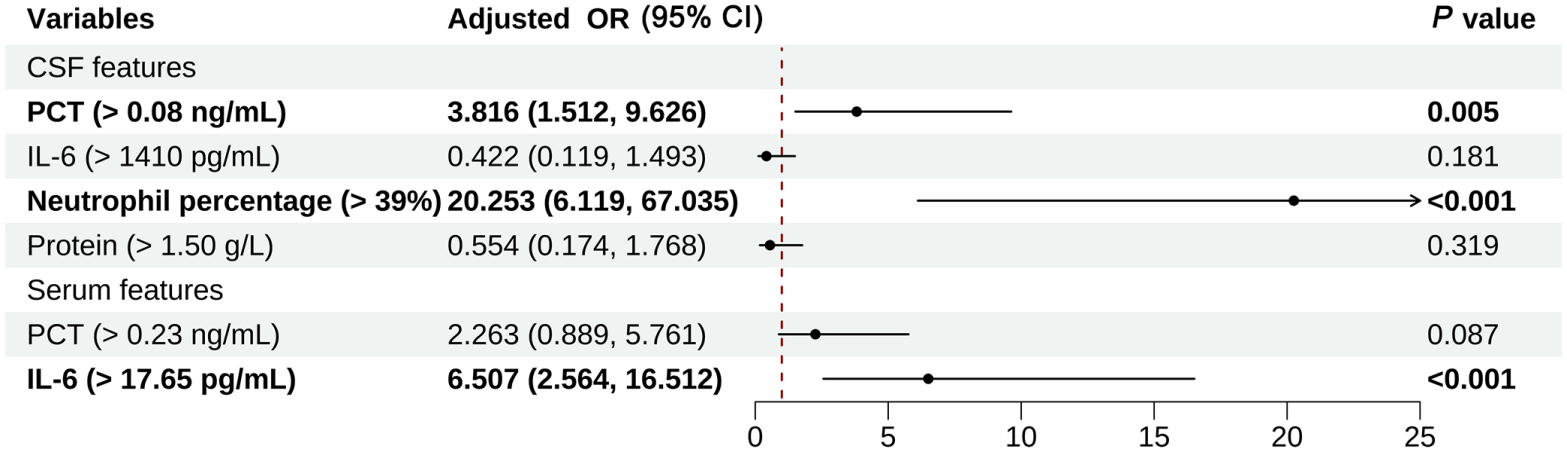
Multivariate analysis of variables for distinguishing acute bacterial meningitis (BM) from tuberculosis meningitis (TBM).

Multiple logistic regression analysis confirmed that elevated CSF-PCT (> 0.08 ng/mL), elevated CSF neutrophil percentage (> 39%), and increased serum IL-6 (> 17.65 pg/mL) were independent predictors for distinguishing BM from TBM (**Figure 2**). Specifically, a CSF-PCT level > 0.08 ng/mL was associated with a fourfold increased risk of BM (OR = 3.816, 95% CI: 1.512 - 9.626), while a CSF neutrophil percentage >39% conferred a substantially elevated risk (OR = 20.253, 95% CI: 6.119 - 67.035) (**Figure 2**).

**Figure 3:**
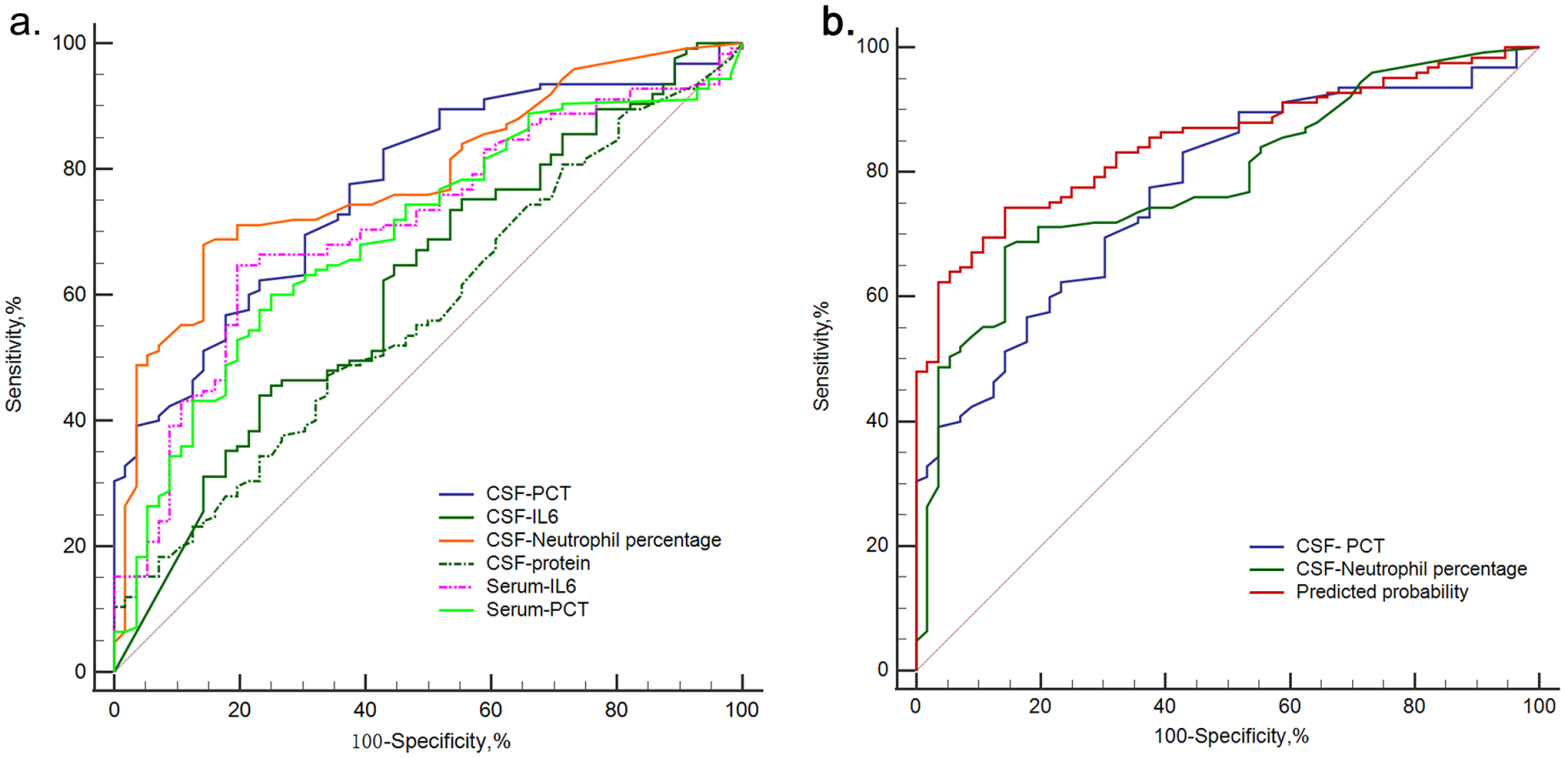
Diagnostic performance of biomarkers for discriminating bacterial meningitis (BM) from tuberculous meningitis (TBM). **(A)** Comparison of Receiver Operating Characteristic (ROC) curves for cerebrospinal fluid procalcitonin (CSF-PCT), CSF interleukin-6 (CSF-IL6), CSF neutrophil percentage, CSF protein, serum procalcitonin (Serum-PCT), and serum interleukin-6 (Serum-IL6). Area under the ROC curve (AUROC) values were: CSF-PCT, 0.772 (95% CI 0.703-0.831); CSF-IL6, 0.617 (95% CI 0.542-0.688); CSF neutrophil percentage, 0.786 (95% CI 0.719-0.843); CSF protein, 0.572 (95% CI 0.496-0.645); Serum-PCT, 0.695 (95% CI 0.622-0.761); Serum-IL6, 0.712 (95% CI 0.640-0.777). **(B)** Comparison of ROC curves for CSF-PCT, CSF neutrophil percentage, and a combined predictor. The combined indicator was derived from a model incorporating CSF-PCT and CSF neutrophil percentage, with the calculation formula: -1.702 + 9.889 × CSF-PCT + 0.030 × CSF neutrophil percentage. The combined indicator achieved an AUROC of 0.849 (95% CI 0.788-0.898).

ROC analysis demonstrated that CSF-PCT distinguished BM from TBM with an AUC of 0.772 (95% CI: 0.703 - 0.831, SE = 0.036). At the optimal cutoff of >0.08 ng/mL, CSF-PCT achieved a sensitivity of 83.20% and a specificity of 57.14%. CSF neutrophil percentage showed comparable discriminative ability (AUC = 0.786, 95% CI: 0.719 - 0.843, SE = 0.035), with no significant difference between the two biomarkers (*P* = 0.763). At its optimal threshold of >39%, the CSF neutrophil percentage demonstrated a sensitivity of 68.00% and a specificity of 85.71%. Compared with the CSF neutrophil percentage, the CSF-PCT had a higher sensitivity and negative predictive value (NPV), but a lower specificity and positive predictive value (PPV). In contrast, other biomarkers exhibited moderate to low diagnostic performance: CSF IL-6 yielded an AUC of 0.617 (95% CI: 0.542 - 0.688, SE = 0.045), CSF protein had an AUC of 0.572 (95% CI: 0.496 - 0.645, SE = 0.045), serum PCT had an AUC of 0.695 (95% CI: 0.622 - 0.761, SE = 0.041), and serum IL-6 had an AUC of 0.712 (95% CI: 0.640 - 0.777, SE = 0.040). Notably, the combined indicator integrating CSF-PCT and CSF neutrophil percentage achieved the highest diagnostic accuracy, with an AUC of 0.849 (95% CI: 0.788 - 0.898, SE = 0.028). This combined model significantly outperformed CSF-PCT alone (*P* = 0.009) and the CSF neutrophil percentage alone (*P* = 0.007), demonstrating superior specificity (85.71%) and PPV (92.10%) compared to individual biomarkers. Complete performance metrics are provided in **Figure 3**; **Supplementary Table 3**.

### Prognosis predictors in patients with BM

Among the 125 patients with BM, 24 (19.2%) experienced unfavorable outcomes (GCS ≤ 8 at discharge). Univariate analysis identified several factors significantly associated with poor prognosis, including lower GCS scores on admission (*P* < 0.001), higher APACHE II scores on admission (*P* < 0.001), complications (impaired consciousness, mechanical ventilation, pneumonia, sepsis, and hydrocephalus) (all *P* < 0.05), hypoalbuminemia (*P* = 0.041), elevated CSF-PCT levels (*P* = 0.001), and increased serum PCT (*P* < 0.001) (**Table 2**).

**Table 2:** Univariate analysis of predictors for favorable versus unfavorable outcomes in patients with bacterial meningitis (BM).

Parameters	Unfavorable outcomes (n = 24) (GCS ≤ 8)	Favorable outcomes (n = 101) (GCS > 8)	*P* value
Demographic profile			
Age, y, IQR^a^	45.5 (36.0, 59.0)	46.0 (29.5, 54.0)	0.358
Gender, male, n (%)^b^	21 (87.5)	73 (72.3)	0.121
Diabetes mellitus, n (%)^b^	5 (20.8)	10 (9.9)	0.163
Clinical feature on admission			
GCS score on admission, IQR^a^	6 (5, 10)	12 (8, 15)	**< 0.001**
APACHE II score on admission, IQR^a^	18 (16, 21)	8 (4, 12)	**< 0.001**
Fever, n (%)^b^	24.0 (100.0)	93.0 (92.1)	0.352
Headache, n (%)^b^	16 (66.7)	74 (73.3)	0.517
Seizure, n (%)^b^	6 (25.0)	27 (26.7)	0.863
Neck stiffness, n (%)^b^	18 (75.0)	76 (75.2)	0.980
Mental symptoms, n (%)^b^	9 (37.5)	36 (35.6)	0.865
Consciousness impairment, n (%)^b^	22 (91.7)	72 (71.3)	**0.038**
Vomiting, n (%)^b^	10 (41.7)	56 (55.4)	0.224
Focal neurological defects, n (%)^b^	10 (41.7)	58 (57.4)	0.164
Status epilepticus, n (%)^b^	0	10 (9.9)	0.207
Treatment			
Mechanical ventilation, n (%)^b^	9 (37.5)	13 (12.9)	**0.014**
Corticosteroid treatment *, n (%)^b^	11 (45.8)	54 (53.5)	0.501
Time to antibiotic initiation, d, IQR^a^	2.0 (1.0, 4.8)	4.0 (1.5, 7.0)	0.146
Duration of empirical antibiotic pretreatment, d, IQR^a^	6.5 (3.3, 13.8)	8.0 (2.0, 15.0)	0.770
Complication			
Pneumonia, n (%)^b^	21 (87.5)	64 (63.4)	**0.023**
Sepsis, n (%)^b^	13 (54.2)	30 (29.7)	**0.023**
Hydrocephalus, n (%)^b^	11 (45.8)	25 (24.8)	**0.040**
Urinary tract infection	4 (16.7)	14 (13.9)	0.749
Laboratory profile			
Albumin, g/L, (mean ± standard deviation (mean ± SD): $\left(\bar{X}\pm s\right)$)^c^	32.38±5.98	34.89±5.19	**0.041**
CSF-PCT, ng/mL, IQR^a^	0.61 (0.15, 1.72)	0.14 (0.09, 0.25)	**0.001**
CSF-IL6, pg/ml, IQR^a^	2877.00 (155.70, 5000.00)	597.10 (61.95, 4474.50)	0.059
Serum-PCT, ng/mL, IQR^a^	2.42 (0.34, 6.04)	0.30 (0.08, 1.03)	**< 0.001**
Serum-IL6, pg/ml, IQR^a^	33.76 (13.73, 61.21)	23.99 (9.35, 69.28)	0.756
Other parameter			
Delay between prodrome and lumber puncture, d, IQR^a^	3.0 (2.0, 6.8)	6.0 (3.0, 10.5)	0.104
PCT time**, d, IQR^a^	10.0 (6.0, 22.5)	13.0 (7.0, 20.0)	0.918
NICU length of stay, d, IQR^a^	17.0 (10.5, 34.0)	15.0 (10.0, 23.5)	0.355

IQR, interquartile range; GCS, Glasgow Coma Scale; APACHE II, Acute Physiology and Chronic Health Evaluation II.

CSF, cerebrospinal fluid; PCT, procalcitonin; IL-6, interleukin-6; NICU, neuro-intensive care unit.

^a^ Mann-Whitney *U* test. ^b^*χ^2^* or Fisher exact test. ^c^Student’s *t* test.

^*^ Corticosteroid treatment including intravenous administration and/or epidural injection. ^**^PCT time was defined as the interval from symptom onset to the first collection of both CSF and serum for PCT measurement in our hospital for each patient, it is equal to the duration of respective disease.Bold values indicated statistically significant differences.

**Figure 4:**
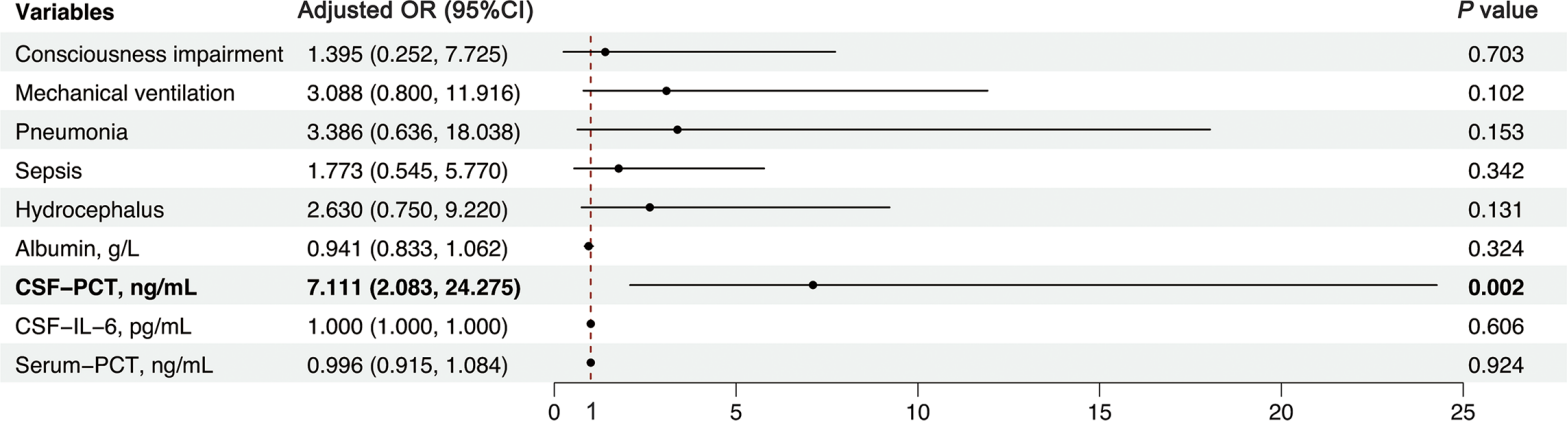
Multivariate analysis of predictors for unfavorable outcomes in patients with bacterial meningitis (BM).

Multivariate logistic regression model identified that the CSF-PCT was the only independent predictor of unfavorable prognosis (adjusted OR = 7.111, 95% CI: 2.083–24.275, *P* = 0.002) (**Figure 4**). ROC curve analysis determined the optimal cut-off value of 0.271 ng/mL for predicting poor outcomes, which yielded an AUC of 0.727 (95% CI: 0.640–0.803), with a sensitivity of 66.67%, a specificity of 77.23%, a PPV of 41.00%, and a NPV of 90.70%.

## Discussion

Our study confirms the superior diagnostic efficacy of CSF-PCT and CSF neutrophil percentage in distinguishing BM from TBM. This advantage is particularly evident in patients who have received empirical antibiotic therapy, outperforming other CSF and serum biomarkers, including serum IL-6 and serum PCT. Furthermore, we have established for the first time that a combined indicator incorporating both CSF-PCT and CSF neutrophil percentage exhibits the best discriminative performance. In prognostic modeling, CSF-PCT has been demonstrated to be an independent risk factor with remarkable predictive ability for unfavorable outcomes in BM. Given their accessibility, rapid turnaround time, and cost-effectiveness, this biomarker combination is well-suited for implementation in resource-limited primary hospitals and can also serve as a valuable adjunct to facilitate timely diagnosis in tertiary hospitals.

The diagnosis of BM continues to pose a significant challenge due to the similarity in clinical features and laboratory examinations with TBM (Paing et al., 2024). Furthermore, the administration of empirical antibiotic therapy often complicates the diagnostic process for BM by altering typical laboratory parameters. A recent systematic review has highlighted the variable impact of previous antibiotic treatment on common diagnostic tests for BM, ranking them from least to most affected as follows: CSF white blood cell count, blood C-reactive protein level, blood PCT level, CSF glucose and protein, and CSF lactate, though CSF-PCT was not evaluated (Ivaska et al., 2024). In many developing regions, such as China, the use of empirical antibiotics is widespread (Currie et al., 2011). In our study, 84.8% of BM patients and 100% of TBM patients had received antibiotic treatments before lumbar puncture. In such circumstances, a biomarker that remains reliable despite prior antibiotic exposure is essential to guide timely and targeted therapy.

CSF-PCT plays a multifaceted role in the pathological mechanisms of BM. The synthesis and secretion of PCT are regulated by the cascade of inflammatory cytokines released during systemic infections (Becker et al., 2010). A key characteristic of PCT is that its production is induced by bacterial infection but remains largely unresponsive to viral challenges, which forms the basis for its utility in discriminating between meningitis types (Sager et al., 2017). This process is promoted by bacterial response cytokines, such as tumor necrosis factor-alpha (TNF-α), but is suppressed by interferon-gamma (IFN - γ) (Maruna et al., 2000; Shi et al., 2022). Conversely, the dominant host defense in TBM is cell-mediated immunity characterized by high levels of IFN - γ (Wilkinson et al., 2017). This mechanistic basis explains why PCT levels are significantly elevated in BM compared to TBM and correlate with disease severity (Kim et al., 2016). Serum PCT is well-established as a valuable biomarker for distinguishing bacterial from non - bacterial meningitis in adults (Velissaris et al., 2018). In contrast, the application of CSF-PCT remains relatively limited. Even though some studies have suggested that it may outperform serum PCT in diagnostic performance (Li et al., 2017), it is important to note that most of these studies were conducted on patients without prior antibiotic treatment. Our group has previously demonstrated that CSF - PCT retains promising diagnostic utility even in patients who have received antibiotics. This current study corroborates this earlier finding. Furthermore, we identified the CSF neutrophil percentage as an additional informative biomarker. Importantly, a combined model incorporating both CSF - PCT and the CSF neutrophil percentage achieved the highest discriminative power in differentiating BM from TBM.

Numerous studies have identified prognostic factors for BM, including concurrent sepsis, systemic compromise, depressed consciousness, and infection with *S. pneumoniae* (van de Beek et al., 2004; Martin-Cerezuela et al., 2023). Consistent with these reports, our initial research also associated impaired consciousness, mechanical ventilation, pneumonia, sepsis, and hydrocephalus with an unfavorable prognosis. Beyond these established factors, we further identified hypoalbuminemia, elevated levels of CSF-PCT, and increased serum PCT levels as potential predictors of adverse outcomes. Multivariate analysis revealed that only CSF-PCT remained an independent risk factor for poor prognosis in BM. At a cutoff value of 0.271 ng/mL, CSF-PCT predicted unfavorable outcomes with a sensitivity of 66.67% and a specificity of 77.23%. In contrast, a previous study reported an association between serum PCT and poor outcome (cutoff value 1.10 ng/mL, sensitivity 75%, specificity 70%) but did not establish it as an independent predictor (Ko et al., 2017). This discrepancy may be attributed to the greater susceptibility of serum PCT to antibiotic therapy (Viallon et al., 2005).

Several limitations should be acknowledged. Firstly, this is a single-center study involving a relatively small population. Secondly, the registration period for this study lasted 11 years. Given the limitations of diagnostic technology over different years, not all cases of BM or TBM had a definitive etiological diagnosis. Thirdly, the predictive ability of CSF-PCT for the long-term prognosis of BM was not investigated. Fourthly, systematic long-term follow-up data were not available for all probable TBM cases, as some patients were lost to follow-up after discharge or transferred back to local hospitals. Future prospective studies with standardized long-term follow-up protocols would be valuable to further validate biomarker performance in this diagnostically challenging subgroup. Lastly, as the majority of patients were referred from multiple primary care institutions, comprehensive records of specific antibiotic agents, dosages, and durations were unavailable for all enrolled patients. Consequently, clinical outcomes and any impact of antibiotic therapy on the biomarkers (CSF-PCT levels and neutrophil percentage) were elusive in our study, prospective studies with standardized antibiotic recording and serial biomarker sampling are warranted to fully clarify this issue. In conclusion, future research requires a multi-center, large-scale cohort with definitive etiological confirmation, and long-term follow-up to validate and expand our findings.

## Conclusion

In conclusion, our findings indicate that the combination of CSF-PCT and CSF neutrophil percentage serves as a reliable biomarker for differentiating BM from TBM. Furthermore, CSF-PCT is an independent predictor of poor outcomes in BM, especially among patients with prior empirical antibiotic treatment. These findings may assist clinicians in the early-stage differentiation of BM from TBM and in determining the short-term prognosis of BM.

## Data Availability

The original contributions presented in the study are included in the article/**Supplementary Material**. Further inquiries can be directed to the corresponding authors.
