# Supplementary material for: Cerebrospinal fluid procalcitonin and neutrophil percentage: a combined biomarker for differentiating bacterial from tuberculous meningitis in antibiotic-pretreated patients
Source: Front Cell Infect Microbiol. 2026 Jun 23;16:1825236. doi: 10.3389/fcimb.2026.1825236 (PMC13337707; doi:10.3389/fcimb.2026.1825236)
Supplement: Supplementary file 1 [file Table1.docx]

**Supplemental Table 1. Bacterial pathogens among 75 acute bacterial meningitis patients.**

| **Pathogens** | **Number of patients** |
| --- | --- |
| Gram-positive bacteria | 7 |
| Gram-negative bacteria | 4 |
| Streptococcus species | 19 |
| *Streptococcus pneumoniae* | 15 |
| *Viridans streptococci* | 1 |
| *Streptococcus suis* | 1 |
| *Streptococcus mutans* | 1 |
| *Streptococcus intermedius* | 1 |
| Staphylococcus species | 14 |
| *Staphylococcus epidermidis* | 6 |
| *Staphylococcus aureus* | 3 |
| *Staphylococcus haemolyticus* | 2 |
| *Staphylococcus caprae* | 1 |
| *Staphylococcus capitis* | 1 |
| *Staphylococcus warneri* | 1 |
| Klebsiella species | 11 |
| *Klebsiella pneumoniae* | 10 |
| *Klebsiella variicola* | 1 |
| Listeria species | 3 |
| *Listeria monocytogenes* | 3 |
| Enterococcus species | 3 |
| *Enterococcus faecalis* | 1 |
| *Enterococcus faecium* | 2 |
| Other species | 14 |
| *Escherichia coli* | 3 |
| *Acinetobacter baumannii* | 3 |
| *Bacteroides fragilis* | 1 |
| *Pseudomonas aeruginosa* | 1 |
| *Sphingomonas paucimobilis* | 1 |
| *Actinobacteria* | 1 |
| *Acinetobacter johnsonii* | 1 |
| *Prevotella oris* | 1 |
| *Actinobacillus* | 1 |
| *Caries-associated Actinomyces* | 1 |
