# Supplementary material for: Cerebrospinal fluid procalcitonin and neutrophil percentage: a combined biomarker for differentiating bacterial from tuberculous meningitis in antibiotic-pretreated patients
Source: Front Cell Infect Microbiol. 2026 Jun 23;16:1825236. doi: 10.3389/fcimb.2026.1825236 (PMC13337707; doi:10.3389/fcimb.2026.1825236)
Supplement: Supplementary file 2 [file Table2.docx]

**Supplemental Table 2. Comparison of clinical characteristics between patients with bacterial meningitis (BM) and tuberculous meningitis (TBM).**

|  | BM (n = 125) | TBM (n = 56) | | *P* value | |
| --- | --- | --- | --- | --- | --- |
| **Clinical characteristics** | | |  |  |  |
| Time from onset to CSF-PCT test, median (IQR), d^a^ | 12.0 (6.5, 20.5) | 21.5 (15.0, 31.5) | | **＜0.001** |  |
| Fever, n (%)^b^ | 117 (93.6) | 51 (91.1) | | 0.544 |  |
| Headache, n (%)^b^ | 90 (72.0) | 49 (87.5) | | **0.022** |  |
| Consciousness impairment, n (%)^b^ | 94 (75.2) | 32 (57.1) | | **0.015** |  |
| Neck stiffness, n (%)^b^ | 94 (75.2) | 43 (76.8) | | 0.818 |  |
| Seizure, n (%)^b^ | 33 (26.4) | 11 (19.6) | | 0.327 |  |
| Mental symptoms, n (%)^b^ | 45 (36.0) | 24 (42.9) | | 0.380 |  |
| Vomiting, n (%)^b^ | 66 (52.8) | 25 (44.6) | | 0.310 |  |
| Focal neurological defects, n (%)^b^ | 68 (54.4) | 39 (69.6) | | 0.054 |  |
| Status epilepticus, n (%)^b^ | 10 (8.0) | 4 (7.1) | | 1.000 |  |
| Hydrocephalus, n (%)^b^ | 36 (28.8) | 17 (30.4) | | 0.831 |  |
| Pneumonia, n (%)^b^ | 85 (68.0) | 27 (48.2) | | **0.011** |  |
| **Treatments** |  |  | |  |  |
| Mechanical ventilation, n (%)^b^ | 22 (17.6) | 11 (19.6) | | 0.742 |  |
| Corticosteroids treatment, n (%)^b^ | 65 (52.0) | 41 (73.2) | | **0.007** |  |
| Duration of empiric antibiotic pretreatment, median (IQR), d^a^ | 7 (2.5, 14.5) | 9 (5.0, 12.0) | | 0.516 |  |

Abbreviations: CSF, cerebrospinal fluid; PCT, procalcitonin; IQR, interquartile range.

^a^Mann-Whitney *U* test. ^b^*χ^2^* or Fisher exact test.
