# Supplementary material for: Cerebrospinal fluid procalcitonin and neutrophil percentage: a combined biomarker for differentiating bacterial from tuberculous meningitis in antibiotic-pretreated patients
Source: Front Cell Infect Microbiol. 2026 Jun 23;16:1825236. doi: 10.3389/fcimb.2026.1825236 (PMC13337707; doi:10.3389/fcimb.2026.1825236)
Supplement: Supplementary file 3 [file Table3.docx]

**Supplemental Table 3. Diagnostic performance of the six biomarkers for distinguishing bacterial meningitis (BM) from tuberculous**

**meningitis (TBM).**

| Biomarkers | AUROC | 95% CI | Cut off | Sensitivity (%) | Specificity (%) | PPV (%) | NPV (%) | SE |
| --- | --- | --- | --- | --- | --- | --- | --- | --- |
| CSF PCT | 0.772 | 0.703 - 0.831 | 0.08 ng/mL | 83.20 | 57.14 | 81.20 | 60.40 | 0.036 |
| CSF IL-6 | 0.617 | 0.542 - 0.688 | 1410 pg/mL | 44.00 | 76.79 | 80.90 | 38.10 | 0.045 |
| CSF neutrophil percentage | 0.786 | 0.719 - 0.843 | 39 % | 68.00 | 85.71 | 91.40 | 54.50 | 0.035 |
| CSF protein | 0.572 | 0.496 - 0.645 | 1.50 g/L | 47.20 | 66.07 | 75.60 | 35.90 | 0.045 |
| Serum PCT | 0.695 | 0.622 - 0.761 | 0.23 ng/mL | 60.00 | 75.00 | 84.30 | 45.70 | 0.041 |
| Serum IL-6 | 0.712 | 0.640 - 0.777 | 17.65 pg/mL | 64.80 | 80.36 | 88.00 | 50.60 | 0.040 |
| CSF PCT + CSF neutrophil percentage^*^ | 0.849 | 0.788 - 0.898 | 0.707 | 74.40 | 85.71 | 92.10 | 60.00 | 0.028 |

Abbreviations: AUROC, area under the receiver operating characteristic curve; CI, confidence interval; PPV, positive predictive value;

NPV, negative predictive value; CSF, cerebrospinal fluid; PCT, procalcitonin; IL-6, interleukin-6.

^*^Combined indicators: the CSF PCT + CSF neutrophil percentage, namely the predicted probability, and the calculation formula of combined indicators = -1.702+9.889×CSF PCT+0.030×CSF neutrophil percentage.
